# Posttraumatic Growth Among Young Women, Comparing Risk and Protective Factors in Sexual Violence Survivors Versus Other Trauma Survivors

**DOI:** 10.1007/s40653-024-00649-y

**Published:** 2024-09-14

**Authors:** Tehila Refaeli, Ela Shir

**Affiliations:** The Charlotte Jack Spitzer Department of Social Work, Ben-Gurion University of the Negev, P.O.B. 653, Beer-Sheva, 8410501 Israel

**Keywords:** Post-traumatic growth, Young women, Emerging adulthood, Self-blame, Shame, Social reactions

## Abstract

This study examined differences between young women who are survivors of sexual violence and young women who are survivors of other traumas in terms of posttraumatic growth (PTG) and possible PTG predictors: personal factors (shame and self-blame) and social factors (social support and social reactions to the traumatic event). Additionally, the study explored the possible association between these factors and PTG among the two groups. The sample comprised 285 female trauma survivors, aged 18–30, of whom 128 were sexual violence survivors. Lower PTG was found among sexual violence survivors, while shame, self-blame, and receiving negative reactions were higher among this group than the other group. Among both groups, higher levels of PTG were associated with low levels of shame and high levels of positive reactions, but only in those who experienced sexual violence was PTG associated with high levels of self-blame. Implications for research and practice are discussed.

## Introduction

The phenomenon of sexual violence against women has been widely exposed in the last decades, and social awareness of it is growing. Sexual violence is defined as coercion of sexual conduct of any kind against people’s will, and conduct not based on their choice, when they are either children or adults (Rozée, 1993; World Health Organization, 2021). The data from the World Health Organization (2021) indicate that one of three women will experience either physical and/or sexual violence at some point in their lifetimes. Sexual violence is particularly common among young women. In the U.S., for example, young women between the ages of 18 and 24 reported the highest levels of rape and sexual assault compared to any other age group (Sinozich & Langton, 2014).

Psychological research about sexual violence referred to its negative effects (E.g. Ghazali et al., 2018; Green & Webster, 2021). However, recent years have seen the development of the posttraumatic growth (PTG) theory, which suggests that positive psychological changes can result from coping with trauma or harsh life events (Calhoun & Tedeschi, 2014). Nonetheless, these notions have been less explored in terms of coping with sexual violence specifically, which is the aim of the current study (see the systematic review by Fayaz (2024). In this study we examined a wide model of factors that can either enhance or jeopardize PTG among young women between the ages of 18–30. We also compared the contribution they make to PTG between young women who experienced sexual violence vs. young women who experienced other types of traumatic or stressful life events (hereafter, other types of traumas/traumatic events).

### Emerging Adulthood

Arnett (2014) called the period between the ages of 18 and 30 “emerging adulthood,” claiming that young people nowadays do not complete their identity formation during their adolescence, but rather experience this process mainly during their 20s. During this period, young people explore their identity in different life domains such as education and employment as well as through their romantic and sexual relationships. The simultaneously challenges embedded in this transition period jeopardize the young adult’s mental health, resulting among others in high rates of depression, anxiety and psychological distress (Arnett et al., 2014; Malla et al., 2018). Studies also indicated long-term effects of traumatic events (mainly child maltreatment) and trauma-related symptoms on mental health, depression and anxiety among young adults (Cohen et al., 2019; Faulkner et al., 2014; Van Vugt et al., 2014). The way young women cope with present or past sexual violence or other traumatic events during this period can have a significant impact on the formation of their identity (Waterman, 2020).

### Posttraumatic Growth and Sexual Violence

Posttraumatic growth is defined as the experience of positive psychological changes resulting from coping with trauma or harsh life events (Calhoun & Tedeschi, 2014); it is not a direct result of the traumatic event, but rather a result of the individual’s struggle to deal with the event in its aftermath (Taku et al., 2008). While traumatic events can undermine survivors’ core beliefs and schemas concerning themselves and the world around them, PTG models highlight the development of new beliefs and schemas about one’s self, one’s surroundings, and the world, all of which is important for survivors dealing with trauma (Calhoun et al., 2010). Posttraumatic growth, it should be noted, can be experienced alongside the stress elicited by the traumatic events as well as posttraumatic symptoms (See the systematic review by Fayaz (2024).

Three main components comprise PTG. The first is a change in trauma survivors’ perception of the self, including a perception of high internal resilience (Calhoun & Tedeschi, 2014). The second is changes in interpersonal relationships, referring to changes in survivors’ sensitivity to others, as well as a feeling of greater closeness to significant others (Calhoun & Tedeschi, 2014). The third is changes in life philosophy: that is, a change in the meaning of survivors’ lives and their priorities, and seeing life as a gift one should make good use of (Taku et al., 2008).

Although many studies have explored PTG following traumatic and/or stressful life events (e.g., Achterhof et al., 2018; Brooks et al., 2019; Cadell et al., 2014), less is known about PTG in the aftermath of sexual violence (Fayaz, 2024; Ulloa et al., 2016). The studies which have explored PTG among survivors of sexual violence primarily explored the existence of PTG, and the dominance of specific PTG components among this group, and confirmed that indeed PTG exists after sexual violence (e.g., Cole & Lynn, 2010; Fayaz, 2024; McElheran et al., 2012). However, there is less data concerning the particular factors that can predict PTG among this group (Kaye-Tzadok & Davidson-Arad, 2016; Kirkner & Ullman, 2020; Ullman, 2014), and our literature review indicated no previous studies comparing the factors that contribute to or jeopardize PTG among young women survivors of sexual violence versus those of other traumas. This study aimed to fill this gap in the literature by using both personal and social factors as possible predictors of PTG.

### Personal Factors Contributing to PTG

**Shame** is an experience of negative self-perception as a result of a perceived social threat, including the fear of others’ judgment, and it is manifested in strong feelings of embarrassment and inferiority (Budden, 2009). Although the feeling of shame is common after many kinds of trauma (Platt & Freyd, 2012; Stotz et al., 2015), it is a primary feeling among survivors of sexual violence (Vidal & Petrak, 2007). It might be a result of the survivor’s perception of failing to identify the potential danger prior to the assault, the feeling of helplessness during the assault, and/or not responding effectively to the assault (Andrews et al., 2000). Only a few studies have examined the connection between this feeling and PTG in survivors of sexual abuse, and the results of these studies have been inconsistent. One study found that higher levels of shame were linked to lower levels of PTG among women of different ages (Cole & Lynn, 2010). However, another study with a similar population which focused specifically on women who had reported the assault to formal services did not find any such association (Rosenthal et al., 2023). In addition, a study conducted with young women did not find any relationship between shame and PTG (Wolfe, 2023).

**Self-blame** is a cognitive process in which the survivor of a traumatic or stressful life event sees the event as being his/her own fault (Janoff-Bulman, 1979). This process is more relevant to survivors of sexual violence because of the invasive nature of the assault and the widespread societal perspective that victims could have avoided the assault via various means (Feiring et al., 2002): in other words, “victim-blaming”, which might be internalized and recast as self-blame.

Although some studies have indicated that high levels of self-blame are related to high levels of distress, low levels of positive life changes, and low levels of PTG (Frazier et al., 2004; Ullman, 2014), a recent study among young women at-risk indicated the positive contribution of self-blame to PTG (Kaye-Tzadok & Davidson-Arad, 2016). There are two possible explanations for this finding. Firstly, it is assumed that some levels of self-blame can help survivors feel a sense of control over their lives. Another explanation is that PTG is a result of acute distress, as proposed by Tedeschi (1999), and self-blame can contribute to this distress. Therefore, it is possible that PTG arises from the intense distress caused by self-blame (Kaye-Tzadok & Davidson-Arad, 2016).

In 1979, Janoff-Bulman identified two types of self-blame related to sexual violence. Behavioural self-blame is more controlled, as it is based on the situation-specific belief that the survivor’s actions led to the attack. On the other hand, characterological self-blame is based on a negative perception that bad things should happen to the survivor because of their character. This type of self-blame is more difficult to change, as it is deeply rooted in the individual’s overall character. Several studies conducted among sexual violence survivors have indicated that characterological self-blame has more severe effects on mental health compared to behavioural self-blame (see Peter-Hagene & Ullman, 2018 for a review). However, we were unable to find studies that explore the effects of these two types of self-blame on PTG. One study focused on interpersonal violence did not find any differences between the two types of self-blame in terms of their contribution to PTG. Additionally, a combined measure of self-blame had a positive association with PTG (Moschella et al., 2018). For our study, we used a tool that examines self-blame in a more general sense, rather than specific to sexual violence. This allows us to gain insights into participants’ general tendency to attribute blame to themselves.

### Social Factors Contributing to PTG

**Social support** is the exchange of resources between two people with the aim of improving the recipient’s well-being (Shumaker & Brownell, 1984). In this study we focused on the contribution of informal support, namely the support provided by non-professional sources, such as family members and friends. These sources are the most relevant ones for young women during their emerging adulthood; they still rely on their parents as a main source of support (Arnett, 2014), but also rely on and consult with friends during this period (Tanner, 2011). We focused on perceived social support, referring to people’s cognitive evaluation of the availability of their social networks to provide various types of support in times of need (Cohen & Hoberman, 1983).

Social support has been found to be a strong predictor of PTG among people who experience a variety of traumatic events (e.g., Brooks et al., 2019; Noy et al., 2014; Schroevers et al., 2010); however, we found only one study showing that friends’ support contributed to better adjustment – compared to support from other sources – among women who experienced sexual violence (Ullman, 1996). One reason for the lack of studies among this group is that women who experience sexual violence tend to report low levels of support (Kimerling & Calhoun, 1994).

The term **social reactions to the traumatic event** refers to reactions that survivors of traumatic events receive from their environment, which are specific to the traumatic event, and therefore different from social support that is more general and consistent (Ullman, 2010). Ullman (2010) indicated three types of social reactions experienced by survivors of sexual violence. *Positive reactions* include emotional, concrete, and informational support which is specific to the sharing of the assault as well as to the way the survivor deals with it. Two types of negative reactions are: turning against and unsupportive acknowledgment. *Turning against* reactions include blaming the survivor or not believing her story, as well as judgmental reactions regarding her behavior during the assault and stigmatizing reactions to her after the event (e.g., as “damaged goods”). *Unsupportive acknowledgment* reactions can be well-intended but harmful in effect, for instance when others try taking control of the survivor after the assault, when they try to take the survivor’s mind off the incident, etc. (Filipas & Ullman, 2001). All three of these reaction types are reported with great frequency by survivors of sexual violence (Filipas & Ullman, 2001). In an earlier study, positive reactions were related to higher PTG, turning against reactions to low PTG, and unsupportive acknowledgment to no effect on PTG among sexual violence survivors (Ullman, 2014). However, the above study did not focus specifically on young adulthood, a developmental period during which identity is developed via relationships with the close social circles.

### The Current Study

The current study aimed to highlight the possibility of growth in the wake of traumatic events and, specifically, after sexual violence. Pursuant to the literature indicating the severe and unique results of sexual violence, as compared to that of other traumas (Kuwert et al., 2014), it is important to identify differences in PTG, as well as differences in factors contributing to PTG, that can be used for interventions with survivors of sexual violence. As such, this study examined whether there were differences in the PTG levels of young women who were sexual violence survivors compared to the PTG levels in survivors of other kinds of traumatic events. The study then examined which factors contributed to PTG in each group separately: that is, personal factors (comprising shame and self-blame) and social factors (comprising social support and social reactions to the traumatic event). The importance of the current study lies in its focus on young adult women, a group that has received less attention in terms of PTG after experiencing sexual violence. Posttraumatic growth during emerging adulthood can help young women with the complex mission of transitioning into adulthood while at the same time coping with trauma. This study could be instrumental in identifying the mechanism that might support and even empower young women who experienced trauma during such a precarious life stage.

Although some studies have indicated that trauma type is important to PTG (Brooks et al., 2019), in this study we decided to refer to sexual violence in general, as we subscribe to the notion that one type of sexual violence should not be prioritized over others (MacKinnon, 2005). This decision was in part also based on a meta-analysis indicating that type of sexual abuse in childhood, survivor’s age, relationship with attacker, and number of assaults were unrelated to the various effects that were subsequently examined (Paolucci et al., 2001).

## Method

### Sample

The study population comprised Israeli young women between the ages of 18 and 30, who were survivors of various traumatic events. The total sample included 285 young women. We classified the participants into two groups. One group experienced sexual violence and possibly other types of trauma (*n* = 128), while the other group consisted of women who survived other types of traumatic events, possibly multiple events (*n* = 157). Among the second group, common events included the death of a loved one (50.6%) and the illness of a loved one (14%). The mean age was 25.44 (SD = 3.3), and the median was 26. A t-test for independent samples found no significant differences in age between the survivors of sexual violence (*M* = 25.46, *SD* = 3.28) and the survivors of other traumas (*M* = 25.43, *SD* = 3.33), t(283) = − 0.09, n.s. Table 1 shows the distribution of the demographic variables between the two groups, indicating no significant differences in native-born vs. immigrant statuses, levels of education, or employment situations. In terms of the number of traumatic events experienced, a t-test for independent samples revealed significant differences. Survivors of sexual violence (*M* = 3.58, *SD* = 1.52) reported experiencing more traumatic events throughout their lives compared to survivors of other traumas (*M* = 2.15, *SD* = 1.27), t(283) = 8.48, *p* < .001)

**Table 1:** Distribution of demographic variables by type of Trauma and chi square tests

		All women		Survivors of sexual violence		Survivors of other traumas		χ^2^
		N	%	N	%	N	%	
Birth country	Israel	251	88.4	109	85.8	142	90.4	1.46
	Other	33	11.6	18	14.2	15	9.6	
Education^a^	Low level	24	8.4	12	9.4	12	7.6	0.27
	High level	261	91.6	116	90.6	145	92.4	
Employment	Yes	108	85.0	136	86.6	244	85.9	0.15
	No	19	15.0	21	13.4	40	14.1	

^a^ High level = high school diploma and above; low level = no high school diploma

### Process

The participants were recruited through online social networks and through online groups, communities, and forums of women at various ages, some of which were specifically focused on women who experienced sexual violence. We posted a message encouraging women to participate if they had experienced a traumatic event during their life; when they expressed interest in participating, they received an anonymous link. A lottery was held to encourage participation in the study, with a pair of movie tickets as the prize.

### Measures

#### Demographic and Background Characteristics

Participants were asked about their age, their country of birth, and their educational and employment status.

#### Traumatic Life Events

The questionnaire of Solomon (1995) includes ten traumatic or stressful life events (hereafter traumatic life events) such as the deaths or illnesses of loved ones, as well as experiences of any sexual violence. The participants were asked to indicate whether they had experienced any of these events in the past, and they were also able to add other traumatic events (the answers were yes/no). This questionnaire was used to separate the participants into two groups: those who had experienced sexual violence and those who had experienced other traumatic or stressful events (such as the death or illness of a loved one). We also calculated a variable that sums the number of traumatic events experienced by each participant.

#### Posttraumatic Growth

The Posttraumatic Growth Inventory was used (Tedeschi & Calhoun, 1996). The questionnaire includes 21 items which examine whether any positive effects resulted from traumatic and difficult experiences in life, including the experience of new possibilities, sense of personal strength, improvement of relationships with others, sense of spiritual change, and appreciation of life. Responses were on a five-point scale ranging from 1 (*I did not experience this change as a result of the crisis)* to 5 (*I experienced this change to a very great degree as a result of the crisis)*. A mean was calculated for all items. The Cronbach’s α was 0.93.

#### Shame and Self-Blame

Were assessed using the State Shame and Guilt Scale (Tangney & Dearing, 2003). This scale examines the level of shame and self-blame that participants tend to experience in their daily lives. The 15 items are divided into eight items of shame and seven items of self-blame with a scale ranging from 1 (*do not feel this way at all*) to 5 (*feel this way very strongly*). The indexes were the mean of the items for shame and self-blame separately, so that a high score indicated a high degree of shame or self-blame. The Cronbach’s α was 0.91 for shame, and 0.87 for self-blame.

#### Social Support

Was assessed via the Multidimensional Scale of Perceived Social Support (MSPSS) developed by Zimet, Dahlem, Zimet et al. (1988). The MSPSS contains 12 statements that measure participants’ perceptions of the social support they receive from family, friends, and from a significant other. Responses were based on a scale ranging from 1 (*never*) to 5 (*all the time*). Due to the high correlation between the support from a significant other and peer support scales (*r* = .64), the latter was removed from the analysis. Cronbach’s alpha reliability values were 0.93 and 0.92 for family and for significant other support, respectively.

#### Social Reactions to the Traumatic Event

This measure was assessed via the SRQ-S Social Reactions Questionnaire Shortened (Relyea & Ullman, 2015), which includes 16 items to explore the reaction that the participant received from her environment after sharing her traumatic experience. The scale examined the frequency of each response on a scale of 1(*never*) to 4 (*always*). An exploratory factor analysis with Varimax rotation revealed two factors. The first factor represents the positive reactions (eigenvalue 5.94). The second factor represents the negative reactions, and includes both the turning against and unsupportive acknowledgment (eigenvalue 2.02) reactions. Two items were loaded onto the two factors and therefore were removed from the analysis. The positive reaction α was 0.64, and the negative reactions α was 0.88.

### Data Analysis

First, chi square and t-tests were conducted to examine the differences between the groups in terms of background, personal, and social factors. Pearson correlations were conducted to verify the relationship between the independent variables and PTG. A three-step hierarchical regression was conducted for each group separately to examine the contribution of demographic, personal, and social factors to predicting PTG, with the demographic factors used as control variables. The assumption of regression analysis of.

absence of multicollinearity was met.

### Ethical Considerations

The study followed the ethical guidelines of the authors’ institution and was approved by their university’ ethics committee. All participants provided informed consent and were assured that their identities would remain confidential. The phone number of one of the researchers was published in the event that any of the participants wished to call and express discomfort as a result of their participation. None of the participants made use of this option.

## Results

To examine whether there were differences between the sexual violence survivors vs. the other trauma survivors in levels of PTG and in variables that might be associated with PTG, t-tests were conducted for independent samples (see Table 2). As shown in the table, survivors of sexual violence reported significantly lower levels of PTG compared to the other group. The analyses also indicated that young women who were survivors of sexual violence reported significantly more feelings of shame and self-blame compared to the other group. They also reported less family support and less support from their significant others. Concerning the social reactions related to the traumatic events, while there were no significant differences between the groups concerning the positive reactions, survivors of sexual violence reported significantly more negative reactions.

**Table 2:** Differences in PTG and Personal and Social Factors—Survivors of Sexual Violence Versus Survivors of Other Traumatic Events

	All women *M (SD)*	Survivors of sexual violence *M (SD)*	Survivors of other traumas *M (SD)*	T
PTG	2.54 (.91)	2.38 (.86)	2.67 (.94)	-2.68**
Shame	2.39 (.84)	2.69 (.88)	2.16 (.74)	5.42***
Self-blame	2.64 (.85)	2.94 (.86)	2.40 (.75)	5.55***
Family support	4.88 (1.77)	4.37 (1.78)	5.30 (1.65)	-4.57***
Significant other support	6.05 (1.24)	5.76 (1.38)	6.29 (1.05)	-3.69***
Positive reactions	2.58 (.85)	2.60 (.82)	2.55 (.89)	.43
Negative reactions	2.00 (.84)	2.30 (.88)	1.76 (.73)	5.47***

**p < .01, ***p < .001

### Correlations of Personal Factors and Social Resources with PTG

To examine the correlations of personal and social variables with PTG, a series of Pearson correlations was conducted (see Table 3). The Pearson correlations revealed that self-blame did not significantly correlate with PTG. However, among young women who survived traumatic events other than sexual violence, negative correlation was found between shame and PTG. For survivors of sexual violence, the correlation between shame and PTG was close to significant. The social support factors did not correlate with PTG. The positive reactions were positively correlated with PTG among the young women in both groups, and the negative reactions were positively correlated with PTG only among the group of women who survived other traumatic events. The findings also revealed a strong connection between shame and self-blame in both groups. Moreover, there was a notable correlation between negative reactions to self-blame and shame, particularly among the survivors of sexual violence.

**Table 3:** Pearson correlation coefficients between PTG, personal and social factors

	PTG	Shame	Self-blame	Family support	Significant other support	Positive reactions	Negative reactions
PTG	-	− 0.21**	− 0.06	01.	12.	***36.	***30.
Shame	16.-	-	***72.	***27.-	**40.-	04.	***36.
Self-blame	03.	***75.	-	***25.-	**22.-	13.	***37.
Family support	03.-	***46.-	***44.-	-	***46.	09.	**26.-
Significant other support	13.	***41.-	***36.-	***40.	-	04.	***37.-
Positive reactions	***43.	05.	*21.	06.	02.	-	***27.
Negative reactions	11.	***50.	***54.	**26.-	***44.-	**27.	-

Black- Survivors of sexual violence; Gray- Survivors of other traumas

*p < .05, **p < .01, ***p < .001

The control variables, including place of birth (Israel vs. other countries), level of education (high school diploma and above vs. lower level of education), and employment were examined in relation to PTG. Given that place of birth and level of education were related to PTG (not shown), they were entered into the regression analyses as control variables. We also checked for any association between the sum of traumatic events and PTG. We found no significant association for the entire sample (*r* = .7, n.s.), as well as among survivors of sexual violence (*r* = .13, n.s.). The association was weak among survivors of other traumas (*r* = .18, *p* < .05). Additionally, a primary analysis indicated that this variable does not contribute to the regression models for both research groups (not shown). Therefore, this variable was not included in the final multivariate regression analyses.

### Multivariate Regression Analyses of PTG

To assess the overall contribution of the personal and social variables to the prediction of PTG, hierarchical regression analyses were conducted separately for survivors of sexual violence and survivors of other traumatic events (Table 4). The variables were entered in three steps. In the first step, demographic-control variables (country of origin and education) were entered. The personal variables (shame and self-blame) were entered in the second step. Social variables (family support, significant other support, positive reactions, and negative reactions) were entered in the third step.

**Table 4:** Hierarchical multiple regression analysis for predicting PTG

	Survivors of sexual violence (n = 128)						Survivors of other traumas (n = 157)					
	*Step I*		*Step II*		*Step III*		*Step I*		*Step II*		*Step III*	
Predictor	*B*	*β*	*B*	*β*	*B*	*β*	*B*	*β*	*B*	*β*	*B*	*β*
Immigration^1^	− 0.14	− 0.06	− 0.07	− 0.03	0.02	0.01	0.52	0.17*	0.47	0.15	0.21	0.07
Education^2^	− 0.14	− 0.05	− 0.19	− 0.06	0.03	0.01	− 0.88	− 0.25**	− 0.87	− 0.25**	− 0.62	− 0.18**
Shame			− 0.43	− 0.44***	− 0.39	− 0.40**			− 0.36	− 0.28**	− 0.36	− 0.28**
Self-blame			0.35	0.36**	0.18	0.18			0.14	0.11	− 0.02	− 0.02
Family support					− 0.08	− 0.17					− 0.01	− 0.02
Significant other support					0.11	0.17					0.10	0.11
Positive reactions					0.41	0.38***					0.26	0.25***
Negative reactions					0.15	0.15					0.43	0.33***
*R* ^2^	0.01		0.09**		0.28***		0.10***		0.14*		0.31***	
Δ*R*^2^	-		0.08**		0.19***		-		0.05*		0.17***	

^1^ 0 = Born in Israel 1 = Immigrant

^2^ 0 = No high school diploma 1 = High school diploma and above

*p < .05 **p < .01 ***p < .001

As shown in Table 4, the demographic variable in the first step contributed significantly to PTG only for the young women who experienced traumatic events other than sexual violence. In the second step, lower shame was associated with higher PTG in both groups, while higher self-blame was associated with higher PTG only among the survivors of sexual violence. In the last step, positive reactions contributed to higher PTG in both groups, and the negative reactions contributed to higher PTG only among the survivors of other traumatic events. The final model explained 28% of the variance in PTG among the survivors of sexual violence, F(8,115) = 5.60, *P* < .001, and 31.2% among the survivors of other traumatic events, F(8,144) = 8.18, *P* < .001.

## Discussion

Although the emerging adulthood period has received a significant amount of research attention, the issue of PTG as a response to sexual violence, and its meaning during this developmental period, has not. Therefore, as far as we know, the current study is the first to combine the theories behind these two constructs – PTG and the period of emerging adulthood – as they pertain specifically to sexual violence survivors. The current study is also the first, as far as we know, to explore a wide model of both personal and social factors as potential predictors of PTG among sexual violence survivors as well as among a comparison group of young women who experienced other traumas. The importance of exploring PTG among young women in the transitional period to adulthood lies in PTG’s potential to contribute to the process of identity formation, which is part of this transition (Arnett, 2014).

The findings indicated that young women who experienced sexual violence reported lower PTG compared to those who experienced other traumas. Some studies have shown that more traumatic symptoms are related to higher PTG. However, other studies, as highlighted in Fayaz’s systematic review in 2024, have yielded conflicting results. Additionally, it is worth noting that the posttraumatic symptoms resulting from sexual violence are severe and can have long-lasting negative effects on individuals’ lives (Kaplan et al., 2007; Littleton et al., 2014). Based on these studies, and on our finding, one can assume that although in general higher symptoms can lead to higher PTG, at some level the symptoms of the trauma may be too intense in ways that limit the possibility for PTG to occur. This assumption was supported by recent literature that indicated a curvilinear association between posttraumatic symptoms and PTG (Fayaz, 2024).

Our findings also indicated higher levels of shame and self-blame among the women who experienced sexual violence. Previous research has already highlighted the extensive negative consequences of sexual violence (Hunehäll Berndtsson & Odenbring, 2020; Kaplan et al., 2007; Littleton et al., 2014). Furthermore, individuals who have experienced sexual violence often experience intense feelings of shame and self-blame. These emotions stem from survivors’ internalizing society’s negative attitudes toward them (Decou et al., 2017; Frazier, 2003). These attitudes are less common toward survivors of other kinds of traumatic events. The higher levels of shame among this group might also be attributable to the silencing of sexuality in general in society; discussions regarding sexuality are related to strong feelings of shame (Byers, 2011) and, as such, speaking about victimization that involves sexuality may give rise to similar feelings of shame. Although we did not specifically examine shame and self-blame related to sexual violence, it is possible that shame and self-blame following such violence can extend to other areas of life and affect one’s general perspectives. Further exploration is needed to investigate this assumption.

The most important findings from the current study are related to the social reactions to the traumatic event, an area that has received almost no previous research attention (Ullman, 2014). Here we found that survivors of sexual violence received more negative reactions: that is, for example, reactions in which survivors are asked to ignore what they experienced, reactions downplaying the survivors’ feelings, or reactions blaming them for the violence they experienced. An explanation for this phenomenon of “victim-blaming” can be found in the “defensive attribution theory” (Walster, 1966). This theory suggests that when people hear about traumatic events to which other people are exposed, they might feel personally threatened; in order to defend themselves against this threat, they blame the victim, because if the traumatic event is a result of the victim’s behavior, they can act in a way that will not lead them to be victims of the same trauma. Although this reaction may in fact serve as a protective function for the people who did not experience trauma, it is clear that such reactions are damaging to the survivors.

Our findings support previous research that suggests a link between high levels of shame and lower levels of PTG (Cole & Lynn, 2010). Conversely, our results differ from those of Rosenthal et al. (2023) and Wolfe (2023). It is important to note that our study focuses on shame as a general feeling, unrelated to traumatic events. This distinction may explain the varying results in the literature. Given the limited research in this area, our findings suggest the need for further exploration regarding whether differences in the treatment of sexual abuse survivors after movements such as #MeToo will result in lower levels of shame among this population. Ultimately, this may contribute to higher levels of PTG.

The most interesting finding concerns the contribution of self-blame to PTG. Although previous studies indicated mixed findings concerning this correlation – namely, that higher levels of self-blame were related to both higher and lower PTG (e.g., Kaye-Tzadok, & Davidson-Arad, 2016; Moschella et al., 2018; Ullman, 2014) – our findings indicated that among young women, higher levels of self-blame contributed to higher PTG, but that this finding was only relevant to the sample who experienced sexual violence. Here, we should stress that we are examining self-blame in general, rather than self-blame specifically related to sexual violence or traumatic events. This concept is more similar to characterological self-blame (Janoff-Bulman, 1979). It is possible that this type of self-blame, which is associated with more severe mental health issues (see, e.g., Peter-Hagene & Ullman, 2018), may also have positive aspects, such as PTG. This explanation aligns with the findings of Kaye-Tzadok and Davidson-Arad (2016), who studied behavioural self-blame, and Moschella et al. (2018), who examined both behavioural and characterological self-blame. Both studies found a positive association between self-blame and PTG. Their explanation suggests that higher levels of self-blame contribute to greater mental distress, and PTG serves as a coping mechanism for managing this distress and negative self-perception (Kaye-Tzadok & Davidson-Arad, 2016; Moschella et al., 2018).

Additionally, although self-blame should not be encouraged, and although high levels of self-blame can lead to negative results, such as a worsening of posttraumatic symptoms (Herman, 2015; Peter-Hagene & Ullman, 2018; Ullman, 2010), it is important to indicate the possible connection between self-blame and locus of control (Ulloa et al., 2016). Sexual violence is related to taking away individuals’ control over their bodies (Bass & Davis, 2002); self-blame can be seen as reflective of an effort to take back the control that has been taken away during the sexual assault, and in this way helps the survivors adjust to their new situation (Herman, 2015). In accordance, perceived control over the recovery among sexual assault survivors was related to higher PTG (Kirkner & Ullman, 2020).

In the current study we found that social support did not contribute to PTG, a finding which does not align with many previous findings among other groups (e.g., Brooks et al., 2019; Noy et al., 2014; Schroevers et al., 2010). There are a few reasons that might account for this finding. First, in this study we examined the availability of perceived support in general and not of specific types. It might be that some types of support, for example practical or emotional support, are more relevant to PTG among survivors of sexual violence. Additionally, as mentioned previously, survivors of sexual violence receive lower levels of support (Kimerling & Calhoun, 1994) than do survivors of other traumas, and this level of support may not be enough to contribute to their PTG. Aligning with this notion, a previous study indicated that lower familial and especially parental support for survivors of sexual violence was harmful, especially given that survivors had expected to receive protection and guidance (Filipas & Ullman, 2001).

A previous study examined whether different social reactions to the traumatic event predicted growth among sexual violence survivors (Ullman, 2014). The current study focused on this issue among a specific group of young women. We found that positive reactions to the traumatic event were the most powerful contributor to changes in PTG, a finding which supports the pioneering findings of Ullman (2014). As the public discourse concerning sexual violence includes judgment, criticism, and “victim-blaming” (Gervais & Eagan, 2017; Naezer & Oosterhout, 2020), the positive reactions seem to hold special meaning for survivors, and are therefore important predictors of PTG. The positive reactions include, for example, assuring the survivor that he/she is a good person: a reaction which contrasts with the “victim-blaming” discourse that survivors tend to hear in the public arena. These positive reactions can be especially important to young women who are dealing with trauma in the period of their identity formation, as these positive and empowering perceptions can be absorbed into their developing identity. Positive social reactions to sexual violence are also associated with survivors’ better physical and psychological health (Sylaska & Edwards, 2014).

Lastly, the findings indicated a significant association between negative reactions and PTG only for women who experienced other types of traumas, and surprisingly, this association was positive. This positive association may be because we found that this group experienced lower levels of negative reactions. Given the types of events they experienced, such as losing loved ones, it is likely that they did not experience turning against reactions (such as blaming the survivor or not believing her story). Instead, it is more probable that they experienced unsupportive acknowledgement reactions (such as trying to take control of the survivor after the event or trying to take the survivor’s mind off the incident). It appears that for women who experienced other types of traumas, reactions that were found to have adverse effects for sexual violence survivors (see the systematic review by Ullman, 2023) can be interpreted differently. Since shame and guilt are not as prevalent in other traumatic events, these negative reactions can even be perceived as empathetic or caring, as they are sometimes well-intended (Relyea & Ullman, 2015). The lack of a significant negative correlation between negative reactions and PTG among survivors of sexual violence may also be explained similarly, as some of the negative reactions may be seen by survivors as well-intentioned, even if they do not contribute to or are not helpful in their healing process. This assumption is supported by previous research by Sigurvinsdottir and Ullman (2015) that found certain types of negative reactions to be associated with lower self-blame, indicating the need to identify different types of negative reactions and their diverse effects. However, further research is needed to validate this assumption.

## Conclusions

The study highlighted that for young women who are survivors of sexual violence it is harder (compared to other types of trauma survivors) to experience PTG, and hence we must learn what contributes to or decreases PTG in this group. The findings indicated that self-blame’s positive contribution to PTG should be further explored, but must be differentiated from shame, which is associated with a decrease in PTG. In addition, given the ubiquity of “victim-blaming” in contemporary society, positive reactions that survivors receive from those in their close circles can make a strong and unique contribution to their PTG.

The overall findings and the literature review suggests two possible processes for achieving PTG that may appear contradictory. One way to achieve PTG after sexual violence is through positive reactions that promote PTG. Another, more complex and less desirable way to achieve higher levels of PTG is by internalizing victim-blaming attitudes from society, which may lead to self-blame (Relyea & Ullman, 2015; Sigurvinsdottir & Ullman, 2015). Subsequently, the distress resulting from feelings of self-blame can trigger PTG as a defence mechanism (Kaye-Tzadok & Davidson-Arad, 2016; Moschella et al., 2018). Further exploration of the second model is warranted in longitudinal studies. Namely, future studies should explore what the mechanism is that promotes self-blame’s positive contribution to PTG, and how in practice this mechanism can be used to promote positive changes in the life of sexual violence survivors.

Limitations of the current study include the sampling method: We used social media to reach the participants and therefore do not know the response rate, nor the characteristics of those who participated vs. those who did not. However, this method allowed us to reach a variety of young women and not – as is common in many other studies –mainly higher-education students. Another limitation was that we did not gather enough participants to represent specific sub-groups, such as immigrants and minorities, who might experience differences in their PTG and in the predictors of PTG. Future studies should specifically explore these groups in order to gain information about the characteristics related to higher PTG among them. In this study, we divided the participants into two groups based on whether they experienced sexual traumas or other types of traumas, and both groups could have experienced more than one traumatic event. Our findings support previous research (e.g., Caetano & Pereira, 2024; Widyorini et al., 2022), indicating that the accumulation of traumatic events is not associated with PTG. However, it is possible that a more complex statistical model could identify which types and combinations of traumatic events are related to high, low, or no PTG. Lastly, we examined general shame and self-blame that are not specific to sexual assaults. While using this scale allowed us to compare the two research groups, it is necessary to revisit the findings regarding sexual violence survivors using a tool specifically designed for assessing assault-related experiences.

This study also highlighted the importance of positive social reactions to PTG. Additionally, we identified a high prevalence of negative reactions experienced by young women who have survived sexual violence. These findings underscore the necessity for interventions aimed at reshaping the discourse surrounding sexual violence.

## Data Availability

Data are available upon request from the corresponding author.

## References

[CR1] Achterhof, R., Dorahy, M. J., Rowlands, A., Renouf, C., Britt, E., & Carter, J. D. (2018). Predictors of posttraumatic growth 10–11 months after a fatal earthquake. *Psychological Trauma: Theory Research Practice and Policy*, *10*(2), 208–215. 10.1037/tra0000286 28594203 10.1037/tra0000286

[CR2] Andrews, B., Brewin, C. R., Rose, S., & Kirk, M. (2000). Predicting PTSD symptoms in victims of violent crime: The role of shame, anger, and childhood abuse. *Journal of Abnormal Psychology*, *109*(1), 69. 10740937 10.1037//0021-843x.109.1.69

[CR3] Arnett, J. J. (2014). *Adolescence and emerging adulthood*. Pearson Education Limited New York.

[CR4] Arnett, J. J., Žukauskienė, R., & Sugimura, K. (2014). The new life stage of emerging adulthood at ages 18–29 years: Implications for mental health. *The Lancet Psychiatry*, *1*(7), 569–576. 10.1016/S2215-0366(14)00080-7 26361316 10.1016/S2215-0366(14)00080-7

[CR5] Bass, E., & Davis, L. (2002). *The courage to heal: A guide for women survivors of child sexual abuse*. Random House.

[CR6] Brooks, M., Graham-Kevan, N., Robinson, S., & Lowe, M. (2019). Trauma characteristics and posttraumatic growth: The mediating role of avoidance coping, intrusive thoughts, and social support. *Psychological Trauma: Theory Research Practice and Policy*, *11*(2), 232–238. 10.1037/tra0000372 29723030 10.1037/tra0000372

[CR7] Budden, A. (2009). The role of shame in posttraumatic stress disorder: A proposal for a socio-emotional model for DSM-V. *Social Science & Medicine*, *69*(7), 1032–1039. 10.1016/j.socscimed.2009.07.032 19695754 10.1016/j.socscimed.2009.07.032

[CR8] Byers, E. S. (2011). Beyond the birds and the bees and was it good for you? Thirty years of research on sexual communication. *Canadian Psychology*, *52*(1), 20–28. 10.1037/a0022048

[CR9] Cadell, S., Hemsworth, D., Quosai, S., Steele, T., Davies, R., Liben, E., & Siden, S., H (2014). Posttraumatic growth in parents caring for a child with a life-limiting illness: A structural equation model. *American Journal of Orthopsychiatry*, *84*(2), 123–133. 10.1037/h0099384 24826928 10.1037/h0099384

[CR11] Caetano, S., & Pereira, H. (2024). The Mediating Effect of Post-traumatic Growth on the relationship between adverse childhood experiences and psychological distress in adults. *Social Sciences*, *13*(5), 262. 10.3390/socsci13050262

[CR71] Calhoun, L. G., Cann, A., & Tedeschi, R. G. (2010). The posttraumatic growth model: Sociocultural considerations. In T. Weiss & R. Berger (Eds.), *Posttraumatic growth and culturally competent practice: lessons learned from around the globe* (pp. 1–14). John Wiley & Sons.

[CR10] Calhoun, L. G., & Tedeschi, R. G. (2014). *Handbook of posttraumatic growth: Research and practice*. Routledge.

[CR12] Cohen, S., & Hoberman, H. M. (1983). Positive events and social supports as buffers of life change stress. *Journal of Applied Social Psychology*, *13*(2), 99–125. 10.1111/j.1559-1816.1983.tb02325.x

[CR13] Cohen, J. R., Thomsen, K. N., Racioppi, A., Ballespi, S., Sheinbaum, T., Kwapil, T. R., & Barrantes-Vidal, N. (2019). Emerging adulthood and prospective depression: A simultaneous test of cumulative risk theories. *Journal of Youth and Adolescence*, *48*(7), 1353–1364. 10.1007/S10964-019-01017-Y 30949796 10.1007/s10964-019-01017-y

[CR14] Cole, A. S., & Lynn, S. J. (2010). Adjustment of sexual assault survivors: Hardiness and acceptance coping in posttraumatic growth. *Imagination Cognition and Personality*, *30*(1), 111–127. 10.2190/IC.30.1.g

[CR15] Decou, C. R., Cole, T. T., Lynch, S. M., Wong, M. M., & Matthews, K. C. (2017). Assault-related shame mediates the association between negative social reactions to disclosure of sexual assault and psychological distress. *Psychological Trauma: Theory Research Practice and Policy*, *9*(2), 166–172. 10.1037/TRA0000186 27607768 10.1037/tra0000186

[CR16] Faulkner, B., Goldstein, A. L., & Wekerle, C. (2014). Pathways from childhood maltreatment to emerging adulthood: Investigating trauma-mediated substance use and dating violence outcomes among child protective services–involved youth. *Child Maltreatment*, *19*, 219–232. 10.1177/1077559514551944 25287053 10.1177/1077559514551944

[CR17] Fayaz, I. (2024). Systematic review of posttraumatic growth from sexual assault in women. *Journal of loss and Trauma*, *29*(3), 291–312. 10.1080/15325024.2023.2254240

[CR18] Feiring, C., Taska, L., & Chen, K. (2002). Trying to understand why horrible things happen: Attribution, shame, and symptom development following sexual abuse. *Child Maltreatment*, *7*(1), 25–39. 10.1177/1077559502007001003 10.1177/1077559502007001003 11838512

[CR19] Filipas, H. H., & Ullman, S. E. (2001). Social reactions to sexual assault victims from various support sources. *Violence and Victims*, *16*(6), 673. 11863065

[CR20] Frazier, P. A. (2003). Perceived control and distress following sexual assault: A longitudinal test of a new model. *Journal of Personality and Social Psychology*, *84*(6), 1257. 10.1037/0022-3514.84.6.1257 12793588 10.1037/0022-3514.84.6.1257

[CR21] Frazier, P., Tashiro, T., Berman, M., Steger, M., & Long, J. (2004). Correlates of levels and patterns of positive life changes following sexual assault. *Journal of Consulting and Clinical Psychology*, *72*(1), 19–30. 14756611 10.1037/0022-006X.72.1.19

[CR23] Gervais, S. J., & Eagan, S. (2017). Sexual objectification: The common thread connecting myriad forms of sexual violence against women. *American Journal of Orthopsychiatry*, *87*(3), 226–232. 10.1037/ort0000257 28493746 10.1037/ort0000257

[CR22] Ghazali, S. R., Chen, Y. Y., & Aziz, H. A. (2018). Childhood maltreatment and symptoms of ptsd and depression among delinquent adolescents in Malaysia. *Journal of Child and Adolescent Trauma*, *11*(2), 151–158. 10.1007/S40653-017-0196-2/TABLES/5 32318145 10.1007/s40653-017-0196-2 PMC7163867

[CR24] Green, K., & Webster, A. (2021). The relationships between childhood abuse and neglect, sub-clinical symptoms of psychosis and self-harm in a non-clinical community sample. *Journal of Child and Adolescent Trauma*, 1–10. 10.1007/S40653-021-00422-5/FIGURES/1 10.1007/s40653-021-00422-5 PMC9360353 35958727

[CR25] Herman, J. (2015). *Trauma and recovery: The aftermath of violence from domestic abuse to political terror* (3rd edition). New York: Basic Books.

[CR26] Hunehäll Berndtsson, K., & Odenbring, Y. (2020). They don’t even think about what the girl might think about it’: Students’ views on sexting, gender inequalities and power relations in school. *Journal of Gender Studies*. 10.1080/09589236.2020.1825217

[CR27] Janoff-Bulman, R. (1979). Characterological versus behavioral self-blame: Inquiries into depression and rape. *Journal of Personality and Social Psychology*, *37*(10), 1798–1809. 512837 10.1037//0022-3514.37.10.1798

[CR28] Kaplan, H., Sadock, B., & Grebb, J. (2007). *Synopsis of psychiatry: Behavioral sciences clinical psychiatry* (10th ed.). Lippincott Williams & Wilkins.

[CR29] Kaye-Tzadok, A., & Davidson-Arad, B. (2016). Posttraumatic growth among women survivors of childhood sexual abuse: Its relation to cognitive strategies, posttraumatic symptoms, and resilience. *Psychological Trauma: Theory Research Practice and Policy*, *8*(5), 550–558. 10.1037/tra0000103 27018919 10.1037/tra0000103

[CR30] Kimerling, R., & Calhoun, K. S. (1994). Somatic symptoms, social support, and treatment seeking among sexual assault victims. *Journal of Consulting and Clinical Psychology*, *62*(2), 333–340. 10.1037/0022-006x.62.2.333 8201071 10.1037//0022-006x.62.2.333

[CR31] Kirkner, A., & Ullman, S. E. (2020). Sexual assault survivors’ post-traumatic growth: Individual and community-level differences. *Violence against Women*, *26*(15–16), 1987–2003. 10.1177/1077801219888019 31802694 10.1177/1077801219888019

[CR32] Kuwert, P., Glaesmer, H., Eichhorn, S., Grundke, E., Pietrzak, R. H., Freyberger, H. J., & Klauer, T. (2014). Long-term effects of conflict-related sexual violence compared with non-sexual war trauma in female World War II survivors: A matched pairs study. *Archives of Sexual Behavior*, *43*(6), 1059–1064. 10.1007/s10508-014-0272-8 24604012 10.1007/s10508-014-0272-8

[CR33] Littleton, H. L., Grills, A. E., & Drum, K. B. (2014). Predicting risky sexual behavior in emerging adulthood: Examination of a moderated mediation model among child sexual abuse and adult sexual assault victims. *Violence and Victims*, *29*(6), 981–998. 10.1891/0886-6708.vv-d-13-00067 25905140 10.1891/0886-6708.vv-d-13-00067

[CR34] MacKinnon, C. A. (2005). *Legal feminism in theory and practice*. Resling.

[CR35] Malla, A., Shah, J., Iyer, S., Boksa, P., Joober, R., Andersson, N., & Fuhrer, R. (2018). Youth mental health should be a top priority for health care in Canada. *The Canadian Journal of Psychiatry*, *63*(4), 216–222. 10.1177/0706743718758968 29528719 10.1177/0706743718758968 PMC5894919

[CR36] McElheran, M., Briscoe-Smith, A., Khaylis, A., Westrup, D., Hayward, C., & Gore-Felton, C. (2012). A conceptual model of post-traumatic growth among children and adolescents in the aftermath of sexual abuse. *Counselling Psychology Quarterly*, *25*(1), 73–82. 10.1080/09515070.2012.665225

[CR37] Moschella, E. A., Turner, S., & Banyard, V. L. (2018). Posttraumatic growth as a mediator of self-blame and happiness in the context of interpersonal violence. *Violence and Victims*, *33*(6), 1088–1101. 10.1891/0886-6708.33.6.1088 30573552 10.1891/0886-6708.33.6.1088

[CR38] Naezer, M., & van Oosterhout, L. (2020). Only sluts love sexting: Youth, sexual norms and non-consensual sharing of digital sexual images. *Journal of Gender Studies*. 10.1080/09589236.2020.1799767

[CR39] Noy, A., Taubman–Ben-Ari, O., & Kuint, J. (2014). Well-being and distress in mothers of two-year-old singletons and twins. *Women & Health*, *54*(4), 317–335. 10.1080/03630242.2014.896441 24617925 10.1080/03630242.2014.896441

[CR40] Paolucci, E. O., Genuis, M. L., & Violato, C. (2001). A meta-analysis of the published research on the effects of child sexual abuse. *The Journal of Psychology*, *135*(1), 17–36. 10.1080/00223980109603677 11235837 10.1080/00223980109603677

[CR41] Peter-Hagene, L. C., & Ullman, S. E. (2018). Longitudinal effects of sexual assault victims’ drinking and self-blame on posttraumatic stress disorder. *Journal of Interpersonal Violence*, *33*(1), 83–93. 10.1177/0886260516636394 10.1177/0886260516636394 PMC5014733 26956436

[CR42] Platt, M., & Freyd, J. (2012). Trauma and negative underlying assumptions in feelings of shame: An exploratory study. *Psychological Trauma: Theory Research Practice and Policy*, *4*(4), 370–378. 10.1037/a0024253

[CR43] Relyea, M., & Ullman, S. E. (2015). Unsupported or turned against: Understanding how two types of negative social reactions to sexual assault relate to postassault outcomes. *Psychology of Women Quarterly*, *39*(1), 37–52. 10.1177/0361684313512610 25750475 10.1177/0361684313512610 PMC4349407

[CR45] Rosenthal, A., Srinivas, T., Gagnon, K., Dmitrieva, J., & DePrince, A. (2023). Trauma appraisals and posttraumatic growth among survivors of sexual assault. *Psychological Trauma: Theory Research Practice and Policy*. 10.1037/tra0001443 36862480 10.1037/tra0001443

[CR44] Rozée, P. D. (1993). Forbidden or forgiven? Rape in cross-cultural perspective. *Psychology of Women Quarterly*, *17*(4), 499–514. 10.1111/j.1471-6402.1993.tb00658.x

[CR46] Schroevers, M. J., Helgeson, V. S., Sanderman, R., & Ranchor, A. V. (2010). Type of social support matters for prediction of posttraumatic growth among cancer survivors. *Psycho-Oncology*, *19*(1), 46–53. 19253269 10.1002/pon.1501

[CR47] Shumaker, S. A., & Brownell, A. (1984). Toward a theory of social support: Closing conceptual gaps. *Journal of Social Issues*, *40*(4), 11–36. 10.1111/j.1540-4560.1984.tb01105.x

[CR48] Sigurvinsdottir, R., & Ullman, S. E. (2015). Social reactions, self-blame, and problem drinking in adult sexual assault survivors. *Psychology of Violence*, *5*(2), 192. 26366320 10.1037/a0036316 PMC4562326

[CR49] Sinozich, S., & Langton, L. (2014). *Rape and sexual assault victimization among college-age females, 1995–2013*. US Department of Justice, Office of Justice Programs, Bureau of Justice Statistics. Retrieved from https://www.bjs.gov/content/pub/pdf/rsavcaf9513.pdf

[CR50] Solomon, Z. (1995). *Coping with war-induced stress: The Gulf war and the Israeli response*. Plenum.

[CR51] Stotz, S. J., Elbert, T., Müller, V., & Schauer, M. (2015). The relationship between trauma, shame, and guilt: Findings from a community-based study of refugee minors in Germany. *European Journal of Psychotraumatology*, *6*(1), 25863. 10.3402/ejpt.v6.25863 26105045 10.3402/ejpt.v6.25863 PMC4478074

[CR52] Sylaska, K. M., & Edwards, K. M. (2014). Disclosure of intimate partner violence to informal social support network members. *Trauma Violence & Abuse*, *15*(1), 3–21. 10.1177/1524838013496335 10.1177/1524838013496335 23887351

[CR53] Taku, K., Cann, A., Calhoun, L. G., & Tedeschi, R. G. (2008). The factor structure of the posttraumatic growth inventory: A comparison of five models using confirmatory factor analysis. *Journal of Traumatic Stress*, *21*(2), 158–164. 10.1002/jts.20305 18404631 10.1002/jts.20305

[CR54] Tangney, J. P., & Dearing, R. L. (2003). *Shame and guilt*. Guilford Press.

[CR55] Tanner, J. L. (2011). *Emerging adulthood: Encyclopedia of adolescence*. Springer.

[CR56] Tedeschi, R. G. (1999). Violence transformed: Posttraumatic growth in survivors and their societies. *Aggression and Violent Behavior*, *4*(3), 319–341. 10.1016/S1359-1789(98)00005-6

[CR57] Tedeschi, R. G., & Calhoun, L. G. (1996). The posttraumatic growth inventory: Measuring the positive legacy of trauma. *Journal of Traumatic Stress*, *9*(3), 455–471. 10.1007/BF02103658 8827649 10.1007/BF02103658

[CR58] Ullman, S. E. (1996). Do social reactions to sexual assault victims vary by support provider? *Violence and Victims*, *11*(2), 143. 10.1891/0886-6708.11.2.143 8933710

[CR59] Ullman, S. E. (2010). *Talking about sexual assault: Society’s response to survivors*. American Psychological Association.

[CR60] Ullman, S. E. (2014). Correlates of posttraumatic growth in adult sexual assault victims. *Traumatology*, *20*(3), 219. 10.1037/h0099402 25379029 10.1037/h0099402 PMC4217480

[CR61] Ullman, S. E. (2023). Correlates of social reactions to victims’ disclosures of sexual assault and intimate partner violence: A systematic review. *Trauma Violence & Abuse*, *24*(1), 29–43. 10.1177/15248380211016013 10.1177/15248380211016013 34008446

[CR62] Ulloa, E., Guzman, M. L., Salazar, M., & Cala, C. (2016). Posttraumatic growth and sexual violence: A literature review. *Journal of Aggression Maltreatment & Trauma*, *25*(3), 286–304. 10.1080/10926771.2015.1079286 10.1080/10926771.2015.1079286 PMC5831550 29503522

[CR63] Van Vugt, E., Lanctôt, N., Paquette, G., Collin-Vézina, D., & Lemieux, A. (2014). Girls in residential care: From child maltreatment to trauma-related symptoms in emerging adulthood. *Child Abuse & Neglect*, *38*(1), 114–122. 10.1016/J.CHIABU.2013.10.015 24262310 10.1016/j.chiabu.2013.10.015

[CR64] Vidal, M. E., & Petrak, J. (2007). Shame and adult sexual assault: A study with a group of female survivors recruited from an East London population. *Sexual and Relationship Therapy*, *22*(2), 159–171. 10.1080/14681990600784143

[CR65] Walster, E. (1966). Assignment of responsibility for an accident. *Journal of Personality and Social Psychology*, *3*(1), 73–79. 5902079 10.1037/h0022733

[CR66] Waterman, A. S. (2020). Now what do I do? Toward a conceptual understanding of the effects of traumatic events on identity functioning. *Journal of Adolescence*, *79*, 59–69. 10.1016/j.adolescence.2019.11.005 31901704 10.1016/j.adolescence.2019.11.005

[CR67] Widyorini, E., Roswita, M. Y., Primastuti, E., & Wijaya, D. A. (2022). The role of resilience towards the correlation between adverse childhood experiences and post-traumatic growth. *The Open Psychology Journal*.

[CR68] Wolfe, G. L. (2023). Examining the potential role of shame, empowerment, and institutional courage in the relationship between sexual assault and both post traumatic stress and post traumatic growth. Available from ProQuest Dissertations & Theses Global. (2866004009). https://ezproxy.bgu.ac.il/login?url=https://www.proquest.com/dissertations-theses/examining-potential-role-shame-empowerment/docview/2866004009/se-2

[CR69] World Health Organization (2021). *Violence against women* Retrieved from https://www.who.int/news-room/fact-sheets/detail/violence-against-women

[CR70] Zimet, G. D., Dahlem, N. W., Zimet, S. G., Farley, G. K., Sawyer, S. M., & Ramirez, M. (1988). The Multidimensional Scale of Perceived Social Support. *Journal of Personality Assessment*, *52*(1), 30–41. 10.1207/s15327752jpa5201_2

